# Segmentation of Extrapulmonary Tuberculosis Infection Using Modified Automatic Seeded Region Growing

**DOI:** 10.1007/s12575-009-9013-0

**Published:** 2009-07-14

**Authors:** Iman Avazpour, M Iqbal Saripan, Abdul Jalil Nordin, Raja Syamsul Azmir Raja Abdullah

**Affiliations:** 1 Department of Computer and Communication, Faculty of Engineering, Universiti Putra Malaysia, 43400, Serdang, Malaysia; 2 Faculty of Medicine and Health Sciences, Universiti Putra Malaysia, 43400, Serdang, Malaysia

**Keywords:** Seeded Region Growing, Segmentation, Dual Modality Imaging, Positron Emission Tomography, Computed Tomography

## Abstract

In the image segmentation process of positron emission tomography combined with computed tomography (PET/CT) imaging, previous works used information in CT only for segmenting the image without utilizing the information that can be provided by PET. This paper proposes to utilize the hot spot values in PET to guide the segmentation in CT, in automatic image segmentation using seeded region growing (SRG) technique. This automatic segmentation routine can be used as part of automatic diagnostic tools. In addition to the original initial seed selection using hot spot values in PET, this paper also introduces a new SRG growing criterion, the sliding windows. Fourteen images of patients having extrapulmonary tuberculosis have been examined using the above-mentioned method. To evaluate the performance of the modified SRG, three fidelity criteria are measured: percentage of under-segmentation area, percentage of over-segmentation area, and average time consumption. In terms of the under-segmentation percentage, SRG with average of the region growing criterion shows the least error percentage (51.85%). Meanwhile, SRG with local averaging and variance yielded the best results (2.67%) for the over-segmentation percentage. In terms of the time complexity, the modified SRG with local averaging and variance growing criterion shows the best performance with 5.273 s average execution time. The results indicate that the proposed methods yield fairly good performance in terms of the over- and under-segmentation area. The results also demonstrated that the hot spot values in PET can be used to guide the automatic segmentation in CT image.

## 1. Introduction

Tuberculosis infection (TB) has been pandemic in countries within the tropical regions for decades [1]. This airborne disease is easily spread through the air in tiny droplets discharged in a cough by a person suffering from active tuberculosis of the lungs known as pulmonary TB. TB can also affect other parts of body such as brain, bones, lymph nodes, kidneys, and skin. Thereby, it will be named as extrapulmonary tuberculosis (EPTB) infection [2].

Positron emission tomography combined with computed tomography (PET/CT) is opening its way in clinical applications, especially in cancer staging and post-therapy surveillance with expansion into infection and inflammation [3-8]. In this dual imaging modality, PET images provide information on metabolic activity of lesions, while CT images provide morphological information. This fusion technique has helped to increase the visual perception of images; however, diagnosing and analyzing these images is time consuming and a great challenge for experts. Furthermore, early lesion recognition will help determining the most effective treatment to be instituted to the patient. The purpose of this work is to introduce new automated segmentation framework which can help in better diagnosis of medical images, utilizing new dual imaging modalities. This framework can be used as part of automatic lesion detection and classification tools.

Since Adams and Bischof introduced seeded region growing (SRG) segmentation [9], the algorithm has been improving, and different growing criteria have been introduced. Approaches, like comparing the pixel to be added with the average intensity of the region grown at each step, maximum pixel value at region boundary, or maximum value of the pixels inside the region, tried to improve segmentation accuracy by controlling overgrowing to homogenous neighboring areas [9,10]. Hojjatoleslami and Kittler introduced a new approach to SRG using average contrast and peripheral contrast to control the growing process and to make it predictable [11]. Mehnert and Jackway improved SRG algorithm to be pixel-order-independent, processing pixels with same value in parallel, using priority queues [12]. Wan and Higgins expanded SRG a bit further and introduced symmetric region growing based on line-by-line processing of the image, making segmentation less sensitive to the selection of initial seeds [13]. All these algorithms require a starting point to begin segmentation procedure, and their authors have considered different routines for this purpose. Here, we are proposing a new way to start segmentation based on the data acquired from another imaging modality.

The problem with most unsupervised region growing algorithms is that it over-grows to homogeneous neighboring areas. On the other hand, in supervised region growing, the user must define the growing criteria (GC) to match image specifications. Therefore, for different images, certain GC will not always guarantee the best results. Here, we have described and proposed different aspects of GC for SRG algorithm to examine which results in better segmentation of organs in CT image using PET image data as starting point for the segmentation procedure.

The remainder of this paper is organized as follows. In **section** 2, proposed GC for SRG algorithm has been defined, and previously introduced GCs have been carefully examined. The segmentation results have been compared using over- and under-segmentation percentages and time complexity of each method in **section** 3. In **section** 4, we then discuss each algorithm's performance based on the acquired results.

## 2. Materials and Methods

In this study, patients were fasted overnight and injected with 18F-fluorodeoxyglucose (18F-FDG) radionuclide 45 min before the scan. Imaging studies were performed using Biograph 6, Siemens Medical Solutions Inc. PET/CT machine. Acquisition time was 3 min per bed position with seven bed positions covering from vertex to the mid-thigh. CT imaging was performed prior to PET imaging with patients in still position. A bolus injection of 100 ml of iodinated contrast media (Omnipaque 300, Amersham Health) was given intravenously. Acquisition parameters for six slices CT were 130 kV, 60 mAs, 0.8 s per CT rotation, 2.5 mm slice thickness, pitch 1.5.

New PET/CT devices come together on a single platform, and the patient will be imaged for both PET and CT at the same position, so there will be little patient movement [14,15]. Both PET and CT images have been registered using cross-correlation and transformed to the position where they are best correlated [16].

**Figure 1:**
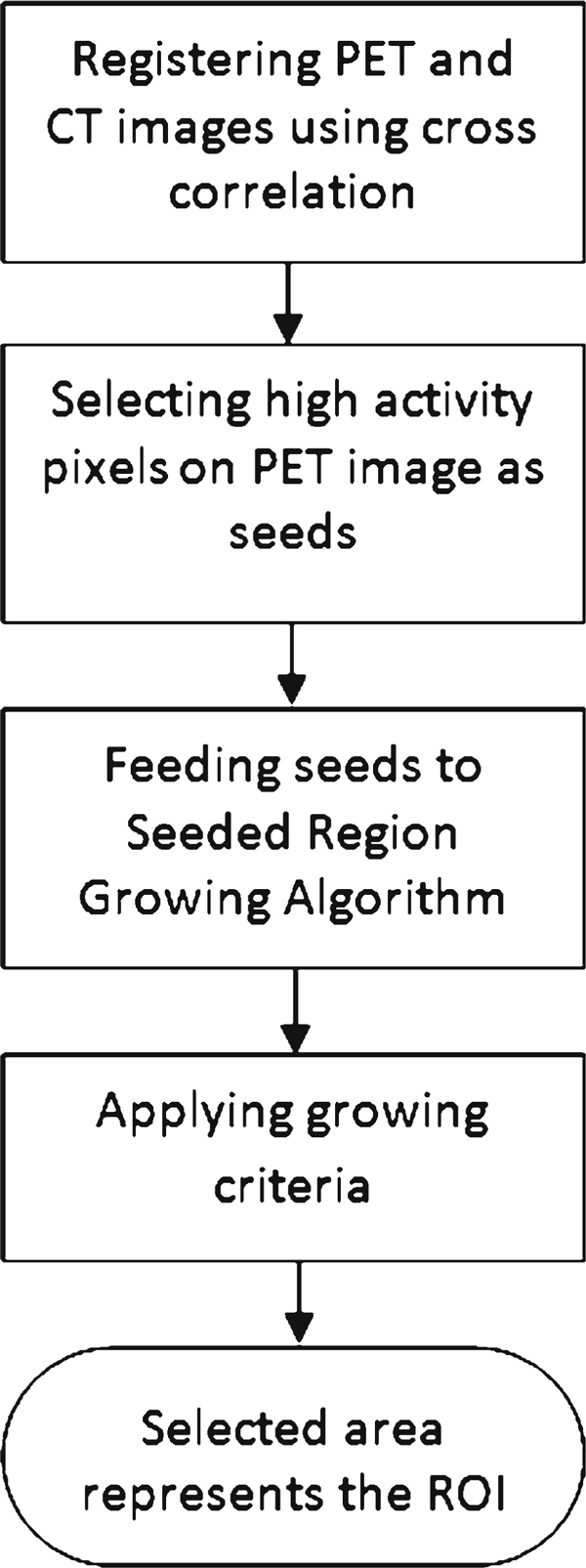
**An overview of the segmentation process**.

High-activity lesions in PET image appear with higher intensity due to more absorption of radio isotope material and higher rate of radioactivity [17]. Malignant cells demonstrate higher metabolic activity than benign lesions. Hence, they absorb more radio isotope material (in this case, 18F-FDG), and the amount of radioactivity will increase in those lesions, causing them to appear brighter in the image [5]. Points with maximum intensity value on PET image were selected as candidates that may suggest infection or malignant lesions. Since candidate points represent the highest pixel intensity, using different brightness and contrast will not affect the procedure. These points will be used to guide segmentation process. An overview of the whole segmentation process has been shown in Figure 1.

**Figure 2:**
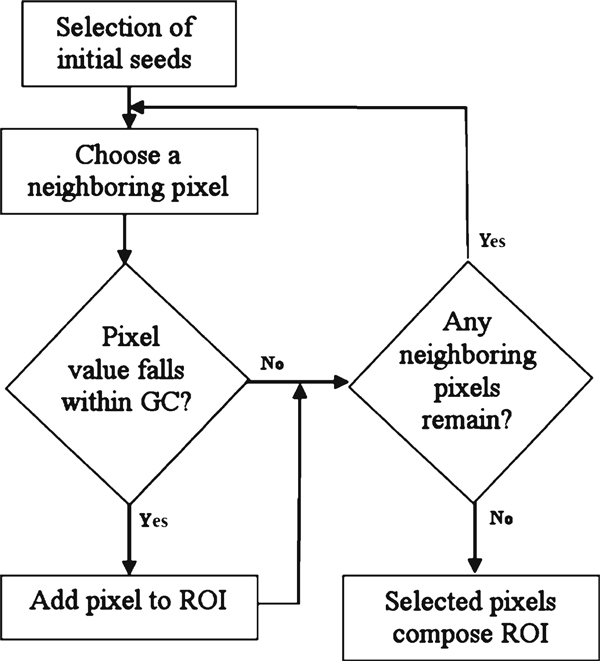
**Seeded region growing algorithm scheme**.

As shown in Figure 1, the focus of our segmentation is on seeded region growing [9]. The algorithm operates by assigning the high-intensity pixel coordinates as starting points of the segmentation procedure and expanding the region of interest (ROI) by checking their neighboring pixels on CT image. A GC will be defined so that at each step boundary, neighboring pixels that fall in this GC will be added to the region. The growing process will continue until there is no other bilinear neighboring pixel of the ROI that falls within this GC. The whole SRG procedure is shown in Figure 2. The method proposed by Adams and Bischof, region averaging [9], has been tested. In addition, we have proposed two other aspects of GC, sliding windows and region averaging and variance.

### 2.1. Region Averaging

Here at each step, the average pixel intensity values of the region grown so far is calculated, and each neighboring pixel's intensity value is compared with this average [9]. We have considered the first seed point as the initial average. As the region grows, the average is calculated to control the growing process. GC has been set to ROI average value ± a threshold value *T*.

(1) GC=Avg(ROI)±T.

Threshold *T* is defined by the user to satisfy image specifications. User is asked to assign a threshold value which has the closest result to desired segmentation.

### 2.2. Local Averaging and Variance

Another aspect is to apply average and variance locally to control growing process. A local mask *M* of 32 × 32 pixels has been used to calculate the average and standard deviation of intensity values in masking area around each seed point. GC then has been defined as:

(2) GC=Avg(M)±STD(M)

Where Avg(*M*) is the average pixel value of the mask *M* and STD(*M*) is its standard deviation. At each step, pixels with the value within this GC will be added to the ROI. Growing process stops when there is no neighboring pixel that satisfies this criterion.

### 2.3. The Proposed Sliding Windows

Computed tomography images have various intensity properties, and different body lesions appear with different intensity; this calls the need for examining the specification of image before segmentation. CT images usually have 512 × 512 pixels dimension, so two local mask *Ms* (16 × 16 pixels) and *Ml* (64 × 64 pixels) centered at the seed point coordinate have been defined and average pixel value of both calculated. Considering these averages, we have:

***If*** Avg(Ms)<Avg(Ml) ***then ****The area to be segmented is brighter than the surrounding area*

***If*** Avg(Ms)>Avg(Ml) ***then ****The area to be segmented is darker than the surrounding area*

***Else ****The segmentation area and surrounding have relatively same intensity*

**Figure 3:**
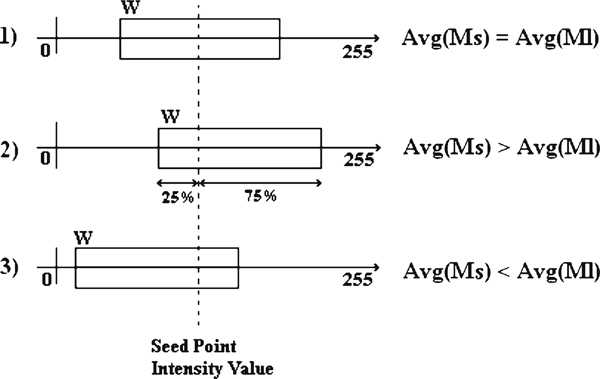
**The concept of sliding window to adapt segmentation criterion**.

Where Avg() is the average pixel value of the masking area. Having grayscale color spectrum, assume GC to be an interval window (*W*) centered at the seed point intensity value. Any pixel with intensity value falling within *W* will be accepted as part of the ROI. If the area to be segmented is relatively of the same average intensity than the surroundings (Avg(Ms) = Avg(Ml)), *W* will be centered at the seed point intensity value. If the area to be segmented is brighter than the surrounding area (Avg(Ms) > Avg(Ml)), *W* will be slid to cover areas of brighter intensity, so we give more credit to brighter pixels to be added to the ROI. The same procedure occurs if the segmentation area is darker (Avg(Ms) < Avg(Ml)), but this time, *W* will be slid to cover areas of darker intensity. This concept is better shown in Figure 3.

The percentage of sliding and the size of masks and *W* can be defined to satisfy the specification of the images to be examined. Here, when there is difference in average intensity of *Ms* and *Ml*, *W* has been slid by 25% of its size. *W* has been defined as a window of size 30. Region growing process will continue until there is no other bilinear neighboring pixel of the ROI with value falling within *W*.

## 3. Results

**Figure 4:**
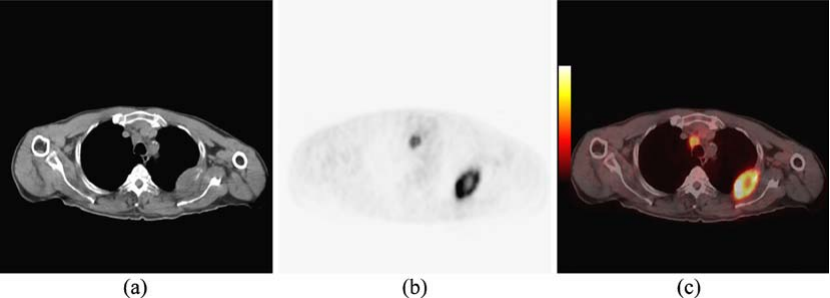
**a CT, b PET, and c PET/CT fused image of a patient having EPTB**.

Images used in this study were acquired using a dual-modality PET/CT imaging device (Biograph 6, Siemens). Fourteen images of patients having EPTB were examined using the above-mentioned algorithms, and all methods were implemented in Matlab. Figure 4 shows CT, PET, and PET/CT fused image of a patient having EPTB. As can be seen in Figure 4a, organ structures are clearly defined in CT image, and boundary edges provide a suitable platform for segmentation algorithms. Figure 4b represents PET image on the same axial cross-section of the patient body. Although areas of high activity can be seen clearly in PET image, it is very difficult to define the body organs they belong to. Therefore, segmentation procedures have been conducted on CT images individually and have not considered the data represented by PET image.

In order to have a basis for segmentation evaluation, images have been sent to medical sources, and desired ROIs have been surveyed and selected manually by a certified radiologist. The manually selected ROIs have been set as benchmark data for optimum segmentation, and the segmentation results have to be compared with this benchmarked data.

**Figure 5:**
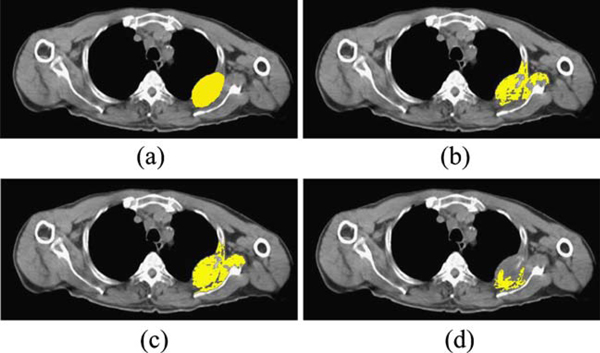
**Segmentation results, a desired manually selected ROI, b SRG using region averaging, c the proposed SRG using sliding windows, d SRG using local averaging and variance**.

Figure 5 shows the results of all segmentation algorithms on the image shown in Figure 4. Manually selected desired ROI is shown in Figure 5a, and segmentation results have been shown in Figure 5b–d. As can be seen visually on images, Figure 5c, which is the segmentation result from the proposed SRG using sliding windows, has the best accuracy in terms of lesion segmentation, but it also deals with some over-segmentation. Figure 5d, which is the result of SRG using local averaging and variance, suffers from under-segmentation. The region has not been grown enough to cover the lesion, and only areas around seed points have been selected.

To evaluate the effectiveness of the proposed methods, segmentation accuracy and time complexity have been considered. Segmentation accuracy has been tested based on calculation of over- and under-segmentation factors. Time complexity also has been defined by measuring the amount of time that each algorithm has consumed to finish its procedure.

The under- and over-segmentation errors were calculated in comparison with the desired manually selected ROI using **Eqs.** 3 and 4.

(3) $$\begin{array}{c} Under\;Segmentation\;f(x,y),\;\overset{⌣}{f}(x,y)=\{(x,y)|(x,y)\\ \in DesiredROI\&(x,y)\notin SelectedROI\} \end{array}$$

(4) $$\begin{array}{c} Over\;Segmentation\;f(x,y),\;\overset{⌢}{f}(x,y)=\{(x,y)|(x,y)\\ \in SelectedROI\&(x,y)\notin DesiredROI\} \end{array}$$

**Figure 6:**
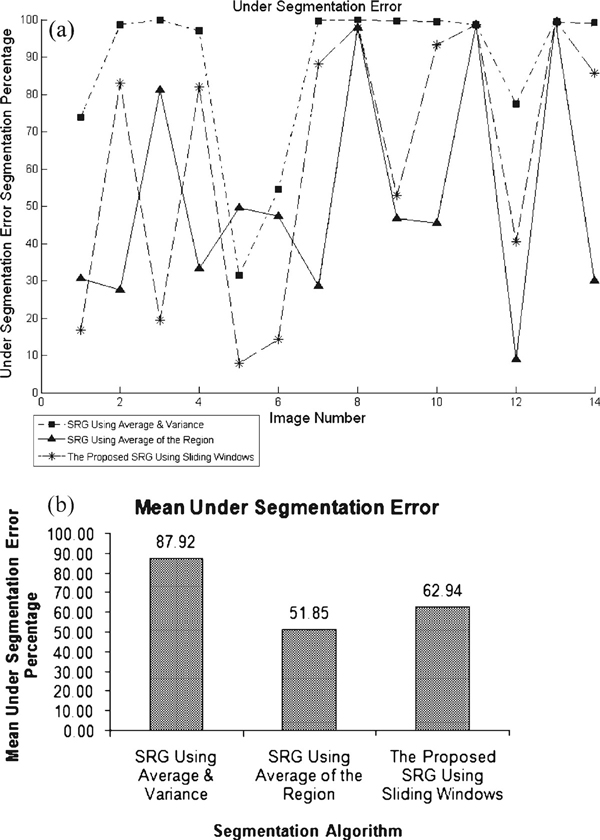
**a Individual under-segmentation error percentage per image and b under-segmentation mean error of all algorithms**.

**Figure 7:**
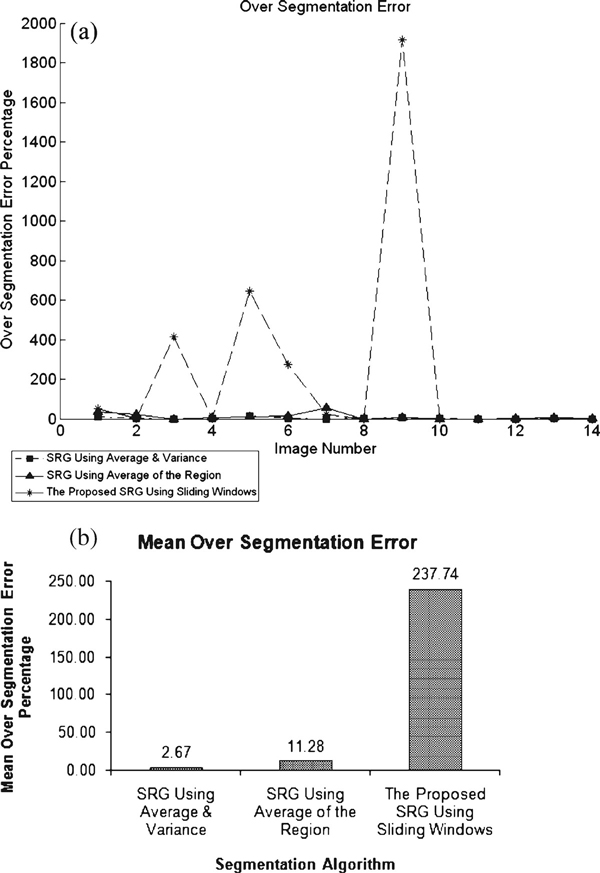
**a Individual over-segmentation error percentage per image and b over-segmentation mean error for all algorithms**.

Where under-segmentation error $\overset{⌣}{f}\;(x,y)$ has been calculated by counting number of pixels (*x*,*y*) in the desired ROI which have not been selected by the algorithm, and the results are shown in Figure 6. Over-segmentation error $\overset{⌢}{f}\;(x,y)$ is also calculated by counting number of pixels (*x*,*y*) in selected area by the algorithm which is not in desired manually selected ROI, and the results are shown by charts in Figure 7. The lesser value indicates better segmentation accuracy.

As can be seen in Figure 6, SRG using local average and variance suffers from under-segmentation and SRG using average of region has the least under-segmentation error. This means that more areas of desired ROI will be covered using SRG with average of the region. On the other hand, considering over-segmentation errors in Figure 7, SRG using local averaging and variance presents the least over-segmentation errors. The proposed SRG using sliding windows deals with over-segmentation.

**Figure 8:**
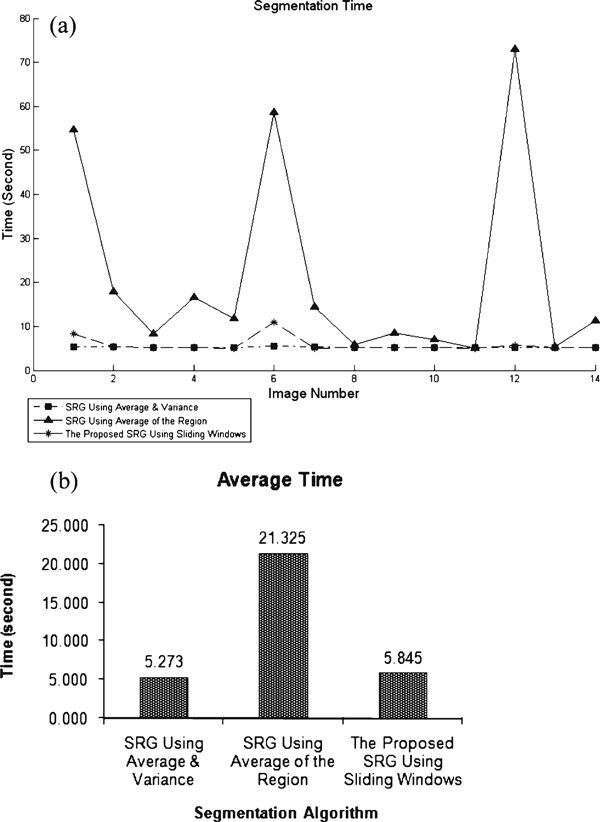
**a Individual time complexity of each algorithm and b average time complexity**.

Since all algorithms have been implemented using the same programming language and they all share the same image registration method, time complexity of the procedure has also been measured and is shown in Figure 8. Since at each step the average of the region grown so far needs to be calculated in SRG using average of the region, the time complexity of the whole process becomes too high when the grown region is relatively big. Other algorithms represent almost the same time complexities.

## 4. Conclusions

Image segmentation is a blind task, and there have been lots of researches to guide segmentation in a way that results in better precision ROI selection. Among segmentation algorithms, region growing highly depends on where the growing process starts and how to control it in order to avoid over-growing to homogenous neighboring areas [13]. Therefore, we proposed the usage of dual modality imaging to use the data acquired from one modality to start the segmentation of images from another modality. Different aspects of GC have been tested to examine the efficiency of seed point selection from PET image on SRG segmentation.

Our effort here was to introduce automated segmentation methods which result in less errors and best performance. Considering the fact that the outputs are to be fed into automatic diagnostic tools, the segmented area must at least represent an estimate of the targeted organ in order for recognition algorithms to be able to recognize it. Therefore, less under-segmentation error is more desirable, bringing the fact that SRG using local averaging and variance cannot offer good results. If time complexity of the process is not an important issue, SRG using average of the region represents the most appreciated performance when guided by PET image data. Otherwise, the sliding windows can be chosen as the GC of choice for SRG segmentation.

This article has proposed a new scheme for automatic segmentation of dual modality medical images using seeded region growing. We proposed a new growing criterion to be used in SRG algorithm and compared its results with previously introduced criterions. Among the methods used here, SRG using region averaging is considered as supervised segmentation since it requires user involvement, and the rest are considered as unsupervised automatic segmentation.
